# Perspective: Growth Monitoring and Promotion as an Opportunity to Improve Early Childhood Development

**DOI:** 10.1016/j.advnut.2025.100470

**Published:** 2025-06-25

**Authors:** Leila M Larson, Edward A Frongillo, Fahmida Akter, Shelbie Wooten, Rebecca L Brander, Marie T Ruel, Jef L Leroy

**Affiliations:** 1 Department of Health Promotion, Education, and Behavior, University of South Carolina, SC, United States; 2 International Food Policy Research Institute, Washington, DC, United States

**Keywords:** growth monitoring and promotion, early childhood development, epidemiology, stunting, psychosocial stimulation, screening

## Abstract

Growth monitoring and promotion (GMP) visits provide a frequent contact point with caregivers, which can be an opportunity for the promotion of early child development (ECD). Using a combination of quantitative analyses of longitudinal and cross-sectional data and a review of the literature, we investigated whether the GMP platform could improve ECD by identifying children at risk of poor development and delivering responsive parenting education to caregivers of young children. Cross-sectional and lagged regression analyses and area under the receiver operating characteristic curves indicated that growth indices were not accurate predictors of concurrent and later child development. Rather, validated tools, such as the Ages and Stages Questionnaire or the Survey of Well-being of Young Children, could be utilized during GMP visits to screen individual children for suboptimal development. Through a review of published literature on ECD interventions, we identified 10 light-touch ECD interventions that could feasibly be implemented during GMP visits, but only half have been evaluated for their effectiveness. Our findings demonstrate that, although growth indices cannot accurately identify children at risk of suboptimal development, the GMP platform could offer an opportunity to screen children for suboptimal development and to deliver ECD interventions. Further evidence on the implementation and effectiveness of light-touch parenting programs, however, is required.

**Statement of significance**

The growth monitoring and promotion platform has the potential to be leveraged to screen children for suboptimal development and deliver light-touch parenting programs to improve early child development using trained frontline health workers.

## Introduction

Growth monitoring and promotion (GMP) is broadly regarded as an essential component of primary health care and includes regular anthropometric measurements (weight, length or height, and head circumference) of infants and young children and subsequent actions if the measurement falls below a certain level. GMP is widely used to screen for nutritional or health problems in children, but there is limited evidence on the effectiveness of GMP to improve the nutritional status of children [[1], [2], [3], [4], [5]].

GMP programs face many challenges in practice, and there is a lack of consensus on the objectives and key components of GMP [[5], [6], [7], [8]]. Recent research on the epidemiological foundations of GMP shows that commonly used GMP criteria do not accurately diagnose or screen for inadequate growth in individual children [9]. Despite these challenges, the frequent contact between caregivers and health workers provides an opportunity to deliver other child-focused services. Given that the GMP program is established as a component of routine preventive child health care in 178 countries globally [10], the GMP platform (that is, infrastructure, staff, established visits) could potentially be used to deliver early childhood development (ECD) interventions by identifying children at risk of poor development and/or delivering responsive parenting education to caregivers of young children in need.

Measures of child height and weight are part of a typical GMP visit. Growth indices have often been associated with ECD outcomes, but their accuracy in identifying individual children at risk of suboptimal development has not been established. Direct measures of ECD are likely to be better at identifying at-risk children, but their use and feasibility within GMP visits remain unexplored. Poor growth and development are, in many cases, reflections of a deprived environment that affects a child’s health, physical and cognitive development, and wellbeing [11].

This study sought to determine *1*) if, and if so, which indicators of growth or ECD assessment tools could be used to identify children at risk of suboptimal mental and motor development (that is, not achieving age-appropriate milestones or growing up in an environment with low quantity and quality of psychosocial stimulation) within the context of GMP; and *2*) what caregiving interventions exist that could feasibly be delivered within GMP visits and what is their effectiveness at improving ECD and psychosocial outcomes. We used 2 large datasets of children living in resource-limited settings to determine the accuracy of using growth indices and indicators to identify children at risk of suboptimal development, and reviewed short and simple ECD screening tools and ECD interventions that could feasibly be incorporated into GMP-type visits.

### The potential for growth indices and indicators to identify children at risk of suboptimal development

Given that growth is already being measured as part of GMP visits, an investigation of whether inadequate growth can be an accurate predictor of poor development is warranted. Many observational studies have shown growth to be associated with child development scores [12]. These associative studies, however, do not provide information on whether growth can accurately predict poor development at the individual level. Evidence on the sensitivity and specificity of inadequate growth as an indicator for targeting individual children at risk of poor development is lacking. To fill this gap, we investigated whether indices of inadequate growth (for example, weight-for-age or height-for-age) can identify individual children at risk of developmental delays.

We first assessed the predictive accuracy of individual-level growth indices and indicators to identify individual children at risk of suboptimal development or at risk of growing up with suboptimal psychosocial stimulation. We then examined whether growth indices may be predictive of child development and psychosocial stimulation at the cluster level, rather than at the individual level, speculating that using growth indices to identify individual children at risk of poor development may not be feasible or accurate in many settings. Moreover, targeting interventions to groups of children, rather than individual children, is likely to be more efficient, feasible, and accurate. Lastly, we explored the accuracy of a combination of growth and other socioeconomic characteristics to predict suboptimal child development.

#### Can repeated or cross-sectional individual-level growth indices and indicators identify children at risk of suboptimal development?

Data from the Maternal and Infant Nutrition Interventions in Matlab (MINIMat) study and the UNICEF Multiple Indicator Cluster Surveys (MICS) were used to assess whether indices of inadequate growth could identify children at risk of developmental delays.

MINIMat study data were used to examine the concurrent and longitudinal associations between height-for-age *z*-scores (HAZ), weight-for-age *z*-scores (WAZ), and weight-for-height *z*-scores (WHZ) and child development scores. The methods for the MINIMat study are described elsewhere [13]. Briefly, MINIMat was a longitudinal study of 4436 pregnant women and their children (3625 live births) living in Matlab, Bangladesh [14]. Children’s weight and height were measured monthly starting at birth until 12 mo of age and quarterly from 12 to 24 mo of age. At 7 mo of age, children’s development was assessed using 2 problem-solving tests (PST, the support and cover tests which assess cognitive development) [15], and the Bayley Scales of Infant and Toddler Development, 2nd Edition (Bayley-II) [16] psychomotor development index (PDI), which measures gross and fine motor development. At 18 mo of age, the Bayley-II’s PDI and mental development index (MDI) were used to assess children’s motor and mental development, and language development was assessed using a language inventory that was developed and adapted based on the MacArthur Communicative Development Inventory [17].

Linear regression models were used to examine the association between HAZ, WAZ, and WHZ at 5, 6, and 7 mo of age, and child development outcomes at 7 mo (that is, PST cover test score, PST support test score, and PDI score). Similarly, linear regression models were used to examine the association among HAZ, WAZ, and WHZ at 12, 15, and 18 mo of age and child development outcomes at 18 mo (that is, PDI, MDI, language comprehension, and language expression). We explored associations with single growth measurements as well as combinations of measurements because previous work [18] suggests that combining more than one growth assessment may yield a more accurate prediction than a single assessment. The root mean square error (RMSE) was used as a measure of inaccuracy for the association between growth indices and child development scores. The RMSE is a measure of the mean difference between the model’s predicted values and the observed values; lower values are indicative of better predictive accuracy. The units are the same as the dependent variable in the model.

Regression analyses between HAZ, WAZ, and WHZ and concurrent and lagged child development outcomes at all ages resulted in wide confidence intervals for the estimate of association (Table 1). For almost all models, the RMSE was large (for example, RMSE = 15.71 for the association between HAZ and motor test scores at 7 mo, meaning that, for an individual close to the mean, the width of the 95% confidence interval for the estimate of association would be 62.8; as reference, the SD was 15.9). The only exception was language expression at 18 mo (RMSE = 0.27 for its association with HAZ or WAZ or WHZ, relative to a SD of 7.4). For all other outcomes, large RMSEs indicate that growth indices are not accurate predictors of child psychomotor or mental development scores at the individual level. Results were similar when using a combination of growth indices from the past 2 to 6 mo.

**TABLE 1:** Estimates of association between growth indices and child development using MINIMat data^1^.

Outcome	*N*	Mean	SD	RMSE	Regression coefficient	SE	RMSE	Regression coefficient	SE	RMSE	Regression coefficient	SE
				HAZ at 7 mo			HAZ at 7 and 6 mo			HAZ at 7, 6, and 5 mo		

Bayley PDI at 7 mo	2090	102.68	15.97	15.71	2.56	0.44	15.70	7 mo: 1.32	7 mo: 0.97	15.66	7 mo: 0.45	7 mo: 1.01
								6 mo: 1.41	6 mo: 0.97		6 mo: -0.26	6 mo: 1.15
											5 mo: 2.70	5 mo: 0.99
Cover test at 7 mo	2090	3.58	1.84	1.85	0.25	0.05	1.85	7 mo: 0.15	7 mo: 0.11	1.84	7 mo: 0.03	7 mo: 0.12
								6 mo: 0.12	6 mo: 0.11		6 mo: -0.09	6 mo: 0.14
											5 mo: 0.35	5 mo: 0.12
Support test at 7 mo	2090	3.06	2.03	2.00	0.25	0.06	2.00	7 mo: 0.10	7 mo: 0.12	2.00	7 mo: 0.04	7 mo: 0.13
								6 mo: 0.17	6 mo: 0.12		6 mo: 0.05	6 mo: 0.15
											5 mo: 0.19	5 mo: 0.13
				HAZ at 18 mo			HAZ at 18 and 15 mo			HAZ at 18, 15, and 12 mo		

Bayley PDI at 18 mo	871	94.13	15.42	15.14	4.7	0.56	15.15	18 mo: 5.16	18 mo: 1.35	15.16	18 mo: 4.82	18 mo: 1.45
								15 mo: -0.50	15 mo: 1.35		15 mo: -0.98	15 mo: 1.54
											12 mo: 0.88	12 mo: 1.38
Bayley MDI at 18 mo	871	77.08	12.33	12.26	3.43	0.45	12.27	18 mo: 3.89	18 mo: 1.09	12.27	18 mo: 4.32	18 mo: 1.17
								15 mo: -0.51	15 mo: 1.09		15 mo: 0.08	15 mo: 1.25
											12 mo: 0.09	12 mo: 1.11
Language comprehension at 18 mo	1043	36.12	7.29	6.56	1.53	0.24	6.55	18 mo: 0.82	18 mo: 0.58	6.55	18 mo: 0.52	18 mo: 0.63
								15 mo: 0.78	15 mo: 0.58		15 mo: 0.36	15 mo: 0.67
											12 mo: 0.78	12 mo: 0.59
Language expression at 18 mo	1043	10.73	7.41	0.27	0.06	0.01	0.27	18 mo: 0.05	18 mo: 0.02	0.27	18 mo: 0.04	18 mo: 0.03
								15 mo: 0.02	15 mo: 0.02		15 mo: 0.01	15 mo: 0.03
											12 mo: 0.01	12 mo: 0.02
				WAZ at 7 mo			WAZ at 7 and 6 mo			WAZ at 7, 6, and 5 mo		

Bayley PDI at 7 mo	2090	102.68	15.97	15.74	2.28	0.42	15.7	7 mo: -1.14	7 mo: 1.31	15.66	7 mo: -1.92	7 mo: 1.35
								6 mo: 3.64	6 mo: 1.31		6 mo: 1.38	6 mo: 1.57
											5 mo: 3.27	5 mo: 1.26
Cover test at 7 mo	2090	3.58	1.84	1.84	0.24	0.05	1.84	7 mo: -0.12	7 mo: 0.15	1.83	7 mo: -0.19	7 mo: 0.16
								6 mo: 0.38	6 mo: 0.15		6 mo: 0.17	6 mo: 0.18
											5 mo: 0.30	5 mo: 0.15
Support test at 7 mo	2090	3.06	2.03	1.99	0.28	0.05	1.98	7 mo: -0.32	7 mo: 0.16	1.97	7 mo: -0.45	7 mo: 0.17
								6 mo: 0.64	6 mo: 0.17		6 mo: 0.24	6 mo: 0.20
											5 mo: 0.58	5 mo: 0.16
				WAZ at 18 mo			WAZ at 18 and 15 mo			WAZ at 18, 15, and 12 mo		

Bayley PDI at 18 mo	871	94.13	15.42	15.33	4.65	0.59	15.34	18 mo: 4.23	18 mo: 1.52	15.32	18 mo: 4.97	18 mo: 1.60
								15 mo: 0.45	15 mo: 1.52		15 mo: 1.73	15 mo: 1.76
											12 mo: –2.02	12 mo: 1.41
Bayley MDI at 18 mo	871	77.08	12.33	12.19	1 3.66	0.47	12.12	18 mo: 0.58	18 mo: 1.20	12.13	18 mo: 0.83	18 mo: 1.27
								15 mo: 3.35	15 mo: 1.20		15 mo: 3.79	15 mo: 1.40
											12 mo: –0.69	12 mo: 1.12
Language comprehension at 18 mo	1043	36.12	7.29	6.56	1.71	0.25	6.57	18 mo: 2.23	18 mo: 0.65	6.57	18 mo: 2.14	18 mo: 0.69
								15 mo: –0.56	15 mo: 0.65		15 mo: –0.70	15 mo: 0.76
											12 mo: 0.22	12 mo: 0.61
Language expression at 18 mo	1043	10.73	7.41	0.27	0.07	0.01	0.27	18 mo: 0.06	18 mo: 0.03	0.27	18 mo: 0.05	18 mo: 0.03
								15 mo: 0.01	15 mo: 0.03		15 mo: 0.002	15 mo: 0.03
											12 mo: 0.01	12 mo: 0.03
				WHZ at 7 mo			WHZ at 7 and 6 mo			WHZ at 7, 6, and 5 mo		

Bayley PDI at 7 mo	2090	102.68	15.97	15.9	0.89	0.44	15.89	7 mo: -0.23	7 mo: 0.80	15.9	7 mo: -0.13	7 mo: 0.83
								6 mo: 1.34	6 mo: 0.79		6 mo: 1.52	6 mo: 0.89
											5 mo: -0.32	5 mo: 0.74
Cover test at 7 mo	2090	3.58	1.84	1.86	0.10	0.05	1.86	7 mo: -0.001	7 mo: 0.09	1.86	7 mo: 0.03	7 mo: 0.10
								6 mo: 0.13	6 mo: 0.09		6 mo: 0.18	6 mo: 0.10
											5 mo: -0.10	5 mo: 0.09
Support test at 7 mo	2090	3.06	2.03	2.01	0.16	0.06	2.00	7 mo: -0.04	7 mo: 0.10	2.00	7 mo: -0.08	7 mo: 0.10
								6 mo: 0.24	6 mo: 0.10		6 mo: 0.16	6 mo: 0.11
											5 mo: 0.14	5 mo: 0.09
				WHZ at 18 mo			WHZ at 18 and 15 mo			WHZ at 18, 15, and 12 mo		

Bayley PDI at 18 mo	871	94.13	15.42	15.73	3.09	0.65	15.73	18 mo: 2.55	18 mo: 1.04	15.73	18 mo: 2.95	18 mo: 1.10
								15 mo: 0.69	15 mo: 1.02		15 mo: 1.22	15 mo: 1.14
											12 mo: -1.01	12 mo: 0.95
Bayley MDI at 18 mo	871	77.08	12.33	12.56	2.63	0.52	12.47	18 mo: 0.67	18 mo: 0.82	12.48	18 mo: 0.50	18 mo: 0.87
								15 mo: 2.48	15 mo: 0.81		15 mo: 2.26	15 mo: 0.90
											12 mo: 0.42	12 mo: 0.75
Language comprehension at 18 mo	1043	36.12	7.29	6.65	1.33	0.28	6.65	18 mo: 1.59	18 mo: 0.44	6.66	18 mo: 1.59	18 mo: 0.47
								15 mo: -0.32	15 mo: 0.43		15 mo: -0.32	15 mo: 0.48
											12 mo: 0.001	12 mo: 0.40
Language expression at 18 mo	1043	10.73	7.41	0.27	0.05	0.01	0.28	18 mo: 0.04	18 mo: 0.02	0.28	18 mo: 0.04	18 mo: 0.02
								15 mo: 0.01	15 mo: 0.02		15 mo: 0.002	15 mo: 0.02
											12 mo: 0.01	12 mo: 0.02

Abbreviations: HAZ, height-for-age z-score; MINIMat, Maternal and Infant Nutrition Interventions in Matlab; MDI, mental development index; PDI, psychomotor development index; RMSE, root mean square error; WAZ, weight-for-age z-score; WHZ, weight-for-height z-score.

1 Units for the mean, SD, RMSE, regression coefficient, and SE are the same as the dependent variables.

As an added measure of predictive accuracy, area under the receiver operating characteristic curves (AUCs) were generated to determine the cut-point of HAZ, WAZ, and WHZ that maximized the sensitivity and specificity to screen for children at risk of developmental delays. Cut-off thresholds of <70, <80, and <85 were used for the Bayley-II PDI index scores at 7 mo and the PDI and MDI index scores at 18 mo of age [19]. Regardless of the cutoffs, AUCs were between 0.5 and 0.6. This confirmed that HAZ, WAZ, and WHZ were inaccurate predictors of PDI and MDI in individual children at either 7 or 18 mo of age (Supplemental Table 1).

Lastly, we examined whether the association between growth indices and child development differed across the distribution of child development scores. On the basis of previous studies [20,21], we might expect that the association between child growth and development is different at the lower end compared with the middle of the distribution. We ran quantile regression models to examine the associations between HAZ, WAZ, or WHZ and PDI or MDI scores at 18 mo of age. Quantile regression estimates indicated that the estimates of association between growth indices and 18-mo PDI scores were higher in the lowest quantile compared with the higher quantiles for PDI scores (Supplemental Figure 1).

The MINIMat study included high-quality and frequently collected data. The study was conducted in Matlab subdistrict, which has slightly better health statistics than other rural areas in Bangladesh, but these differences are small and immaterial to our conclusions.

Multiple Indicator Cluster Survey, 4th round (MICS4) data were taken from a previously combined dataset of cross-sectional survey data from 32 countries [22]. The Early Child Development Index (ECDI) was used to measure literacy-numeracy, physical, socioemotional, and learning development in children aged 3–5 y. The Family Care Indicator (FCI) score was used to assess psychosocial stimulation [23], using the following domains: the availability of playthings and books in children aged 0–5 y, and fathers’ engagement with children and adequacy of care (that is, not left alone or in the care of another child aged under 10 y for >1 h) in children aged 3–5 y [23]. The FCI is strongly associated with child development scores [23]. Height and weight of children were measured at the same time as the ECDI and FCI.

Linear regression models were used to examine the association between HAZ, WAZ, and WHZ and each of the child development and psychosocial stimulation measures, that is, all ECDI domains and FCI domains at the individual level. The RMSE was calculated. AUCs were generated to determine a cut-point of HAZ, WAZ, and WHZ that maximized sensitivity and specificity to identify individual children at risk of low ECDI. Thresholds for adequate ECDI were defined as ≥2 out of 3 on literacy numeracy, and social–emotional subtests, and ≥1 out of 2 on the learning and physical subtests. The threshold for adequate total ECDI was ≥3 out of 10 (on the sum score of all subtests) [24].

Individual-level linear regression models of the association between HAZ, WAZ, WHZ, and domains of ECDI and FCI resulted in large RMSEs, again indicating that growth indices did not accurately identify individual children at risk of suboptimal child development or suboptimal psychosocial stimulation (Table 2). Similarly, AUCs suggested that individual-level growth indices did not discriminate between individual children who are or are not at risk of low ECDI (all AUCs between 0.50 and 0.63) (Supplemental Table 2).

**TABLE 2:** Estimates of individual-level associations between growth indices and child development domains and Family Care Indicator domains, using MICS4 data.

Dependent variable (scores on):	Independent variable: HAZ							Independent variable: WAZ					Independent variable: WHZ				
	*N*	Mean	SD	RMSE	Regression coefficient	SE	*P* value	SD	RMSE	Regression coefficient	SE	*P* value	SD	RMSE	Regression coefficient	SE	*P* value
Literacy numeracy (range: 0–3)	76,007	0.19	0.39	0.39	0.041	0.001	<0.0001	0.39	0.39	0.042	0.001	<0.0001	0.39	0.39	0.007	0.001	<0.0001
Learning (range: 0–2)	75,136	0.84	0.37	0.36	0.031	0.001	<0.0001	0.37	0.36	0.040	0.001	<0.0001	0.37	0.36	0.017	0.001	<0.0001
Social–emotional (range: 0–3)	76,016	0.74	0.44	0.44	0.019	0.001	<0.0001	0.44	0.44	0.022	0.001	<0.0001	0.44	0.44	0.009	0.001	<0.0001
Physical (range: 0–2)	75,053	0.96	0.19	0.19	0.007	0.000	<0.0001	0.19	0.19	0.007	0.001	<0.0001	0.19	0.19	0.000	0.001	0.4468
Total ECDI (range: 0–10)	73,238	0.67	0.47	0.46	0.047	0.001	<0.0001	0.47	0.46	0.056	0.001	<0.0001	0.47	0.47	0.019	0.001	<0.0001
Play things at home (range 0–3)	194,742	0.44	0.50	0.50	0.024	0.024	<0.0001	0.50	0.50	0.030	0.001	<0.0001	0.50	0.50	0.010	0.002	<0.0001
Books at home (range 0–1)	194,742	0.10	0.30	0.35	0.061	0.001	<0.0001	0.30	0.35	0.071	0.001	<0.0001	0.30	0.36	0.024	0.001	<0.0001
Father engagement in activities with child (range: 0–6)	194,747	0.09	0.29	0.42	0.009	0.001	<0.0001	0.29	0.42	0.011	0.001	<0.0001	0.29	0.42	0.004	0.001	0.0032
Adequate care (range 0–2)	191,560	0.75	0.43	0.46	0.049	0.001	<0.0001	0.43	0.46	0.066	0.001	<0.0001	0.43	0.47	0.034	0.001	<0.0001

Abbreviations: ECDI, Early Child Development Index; HAZ, height-for-age z-score; RMSE, root mean square error; WAZ, weight-for-age z-score; WHZ, weight-for-height z-score.

^1^ Units for the mean, SD, RMSE, regression coefficient, and SE are the same as the dependent variables. The score for playthings at home ranged from 0 to 3 and included 1 point for any homemade toys, 1 point for any toys from a shop, and 1 point for any household or outside objects. The score for books at home was categorized as 0 or 1 depending on whether the child had 3 or more children’s books. Father engagement in activities with child was scored out of 6 depending on their engagement in reading books with the child, telling stories, singing songs, taking the child outside, playing with the child, and naming, counting, or drawing things with the child. Adequate care was scored from 0 to 2 based on whether the child was never left alone for >1 h and never left with another child for >1 h.

#### Can cluster-level growth indices and indicators identify groups of children at risk of suboptimal development?

We also tested whether growth indices were predictive of child development at the cluster level, to determine whether growth indices can be used to identify populations in need of ECD interventions. We reasoned that, in certain settings, identifying and targeting communities for ECD interventions would be more feasible than screening and targeting individual children. Using the MICS4 data, we therefore ran linear regressions to examine associations between cluster mean HAZ, WAZ, and WHZ and cluster proportion of children with adequate ECDI domain scores. In MICS4, clusters are areas of ∼80–120 dwelling units. We repeated the above analyses using the cluster proportion of stunting, wasting, and underweight as independent variables rather than cluster mean growth indices. On the basis of the magnitude of the RMSEs of the cluster-level regression models, cluster means for HAZ, WAZ, and WHZ were not accurate predictors of the cluster proportion of children with adequate ECDI (Supplemental Table 3). RMSEs were also large for the models examining associations between the cluster proportion of stunting, wasting, and underweight with the cluster proportion of inadequate ECDI (Supplemental Table 4).

#### Can a combination of growth and socioeconomic characteristics identify children at risk of suboptimal development?

We might expect that a combination of characteristics, rather than one characteristic alone, will be more accurate in predicting ECD, similar to findings from associative studies [25]. From the context of a GMP program, using typically measured demographics such as wealth or education levels in addition to growth to guide the identification of children at risk of poor ECD could be feasible if useful. Using MICS4 data, we examined whether a combination of cluster-level variables could accurately predict cluster-level risk of poor development, compared with a single cluster-level mean growth index. We used multivariable regression models with cluster mean HAZ, WAZ, maternal education, and a household wealth index as independent variables (Supplemental Methods). We included maternal education and household wealth because they are typically and easily collected. We also examined a combination of the cluster proportion of stunting, underweight, and mean maternal education and household wealth index as independent variables. Dependent variables were the cluster proportion of children with adequate ECDI domain scores and the cluster proportion of children with adequate FCI domain scores. Using a combination of cluster-level characteristics (that is, cluster mean HAZ, WAZ, maternal education, wealth index) to predict cluster-level ECDI did not improve the model RMSEs enough to accurately predict cluster-level risk of suboptimal development (Supplemental Tables 5 and 6).

### Using screening tools to identify children at risk of suboptimal development

As an alternative to indices of growth, we tested whether short and simple screening tools to identify children at risk of developmental delay could be incorporated within the context of a GMP visit. We used 2 carefully conducted published reviews [26,27] to identify early child development measurement tools that are feasible to implement, open access, and which could be used to screen individual children for developmental risk during GMP visits by trained health workers. We then conducted a review of peer-reviewed publications reporting on child development screening tools that had the following characteristics: minimum assessment time and assessor skill level required, whether and how the tool was validated, and in which context it has been used.

Developmental screening tools for use by frontline health workers in resource-limited settings have been previously reviewed [27]. Furthermore, the World Bank published a toolkit for measuring early child development in low- and middle-income countries, which details individual-level assessments of ECD [26]. From these publications, we found 4 validated individual screening tools that could be implemented by trained frontline health workers in a short time during GMP-like visits: the Ages and Stages Questionnaire (ASQ), the Survey of Well-being of Young Children (SWYC), the Parents’ Evaluation of Development Status (PEDS), and the PEDS-Developmental Milestones. ASQ is a screening tool to identify developmental delays in children aged 1–66 mo. The ASQ Third Edition assesses domains of communication, gross and fine motor, problem solving, and personal–social development. This parent-report questionnaire can be completed in 10–15 min, which makes it a feasible option for GMP settings. It has been used in over 23 different countries, including by frontline health workers trained in the use of ASQ in many low- and middle-income countries [28]. Furthermore, it has been validated for individual assessment against the Bayley Scales of Infant and Toddler Development and the Differential Ability Scales with high sensitivity and specificity (>85% for both) in children under 24 mo [29]. The second candidate tool, the SWYC, is a 10–15-min parent-report questionnaire used in children aged 2–60 mo, which includes questions on developmental milestones, a pediatric symptom checklist, parental concerns, family risk factors, and emotional changes of the mother. It has been validated against the ASQ and the Bayley Scales of Infant and Toddler Development with good sensitivity and specificity (>70%) [[30], [31], [32]]. Third, the PEDS uses open-ended questions and Likert scales to assess parents’ concerns on their child’s expressive and receptive language, fine and gross motor skills, behavior, social–emotional, self-help, learning, and physical health. It can be used in children from birth to <8 y of age and takes <10 min to administer. Validation studies have determined its accuracy in detecting individual children at moderate or high risk of developmental problems with sensitivity and specificity above 60% [[33], [34], [35], [36], [37]]. Last, the PEDS-Developmental Milestones is a 5-min parent-report questionnaire which can be used with or without the PEDS to measure developmental abilities in the same domains as the PEDS. It has shown good validity with sensitivity and specificity above 80% for the detection of children at psychosocial risk (that is, likely to be delayed, retained in grade, nominated for remedial classes, or dropped out of school) [38].

In summary, although growth indices cannot be used to identify children at risk of suboptimal development, several development screening tools are available to screen children, and these tools are short, valid, include caregiver-reported items, and can be administered by trained frontline health workers, all characteristics that make them feasible to add to GMP-type visits.

### Parenting interventions with the potential to be integrated into GMP visits

Given the important potential for leveraging GMP visits to deliver ECD interventions, we reviewed the literature to identify parenting interventions that could feasibly be delivered within GMP and identified potential adaptations. Our review aimed to identify light-touch versions of existing psychosocial stimulation interventions that could feasibly be delivered by health workers during GMP visits. Light-touch interventions were defined as those that could be delivered by current frontline health workers, volunteers, or healthcare professionals, require <30 min of the caregiver’s time, and require infrequent contact (that is, ≤4 contacts over 12 mo). Through a review of published peer-reviewed and grey literature and through discussions with 2 experts in the field of nutrition and ECD in resource-limited settings, we examined the characteristics of the potential psychosocial stimulation interventions (that is, group compared with individual sessions, home compared with clinic-based sessions, number of visits, adaptation requirements and other relevant information to determine the efficacy of the parenting intervention).

We identified 10 light-touch ECD interventions that could be incorporated into GMP-type visits (Table 3) [[39], [40], [41], [42], [43], [44], [45], [46], [47], [48], [49], [50], [51], [52], [53], [54], [55]]. Many of these interventions include the promotion of age-appropriate play and the use of homemade toys, which makes them suitable for resource-limited settings. Of the 10 interventions identified in this review, only 4 have been evaluated for their effectiveness on child development.

**TABLE 3:** Light-touch early childhood development interventions.

Program	Country	Organizations involved	Intervention delivery location	Target age	Description of the intervention	Screening of children	Evaluation(s) of the intervention
GMP+	Vanuatu	Save the Children Vanuatu Ministry of Health Ministry of Education and Training Vanuatu Society for People with Disability Ministry of Justice and Community Services	Healthcare facility At GMP visits	6 wk to 5 y	Delivery agent: health workers at GMP visit. Program description: GMP+ offered routine contact points to monitor growth and provide encouragement to caregivers on child’s diet and small doable actions tailored to their child’s development. Program was added to routine GMP sessions in 10 selected Health Facilities in Shefa and Sanma Provinces.	Includes developmental milestone assessments to screen children for developmental delays or disabilities aimed at improving early detection, support, and referral.	-
CRED + CCD	Peru	UNICEF Government of Peru	Health facilities for GMP (or CRED in Peru)	Birth to 5 y	Delivery agent: incorporated into every level of their system; CRED nurses delivered content. Program description: system-wide delivery of CCD approach with multisectoral involvement (health, education, social development, protection sectors). Counseling included the following: encourage play and child–caregiver interactions, recommend elements of responsive and sensitive feeding, treat children as respectable individuals, communicate with children affectionately although maintaining eye contact, empower caregivers to provide nurturing care overall. Appointments including CCD approach lasted 30–45 min. CCD has been incorporated into the new standards of the CRED service (in 2018) by officials of the Child's Life Stage programme. The program has become part of training and incorporated into CRED. CRED nurses use CCD approach to counsel caregivers at all CRED appointments.	-	-
Playboxes	Mozambique	Program for Appropriate Technology in Health (PATH) Mozambique Ministry of Health Support from community-based organizations	Healthcare facility waiting room	Birth to 5 y	Materials: Playbox with homemade picture books and toys, safe household objects, poster of developmental milestones Delivery agent: community volunteers Program description: low-cost playbox intervention in 10 health facilities in Maputo Province beginning November 2014. The toy box was placed in the health facility waiting room. Community volunteers were there to engage parents with toys and walk through a simple developmental milestone poster. Parents were encouraged to pick out toys and play with their children, after washing their hands. The community volunteers counseled parents who struggled to interact with their child. On select days, the community volunteers led activities on how to make homemade toys.	-	Karuskina-Drivdale et al 2019 [39] (Mozambique): Design: 1-group pretest–posttest design. Population: caregivers, clinicians, and community volunteers. Assessments: qualitative interviews and focus group discussion. Results: - Improved waiting experience and increased return for follow-up. - Increased caregiver awareness and knowledge of developmental milestones. - Increased stimulation of children by the caregiver. - Increased caregiver engagement during health facility consultations. - Increased detection of developmental problems.
Sit Down and Play (SDP)	India, United States	University of Illinois at Chicago Women’s and Children’s Health Research Unit, Jawaharlal Nehru Medical College	Healthcare facility waiting room or examination room At well-child visits At immunization visits	2–6 mo	Materials: age-specific toys including stackable rings, a set of 3 balls, a wooden car, handout of at home play ideas. Delivery agent: non-professionals or volunteers. Program description: SDP is designed to be a short, low-cost program that incorporates key constructs of social cognitive theory to encourage positive parenting behaviors through take-home play activities. Includes 10 min sessions although families wait in the examination room or in waiting area. During each 10-min session the volunteer: (1) modeled examples of how to use simple age-specific toys to facilitate talking and playing with a child; (2) engaged caregivers in discussions regarding their child’s current developmental abilities and the importance of talking, playing, and interacting with their child; (3) observed caregivers using the toy to play with their child and provided feedback, which emphasizes praising and reinforcing positive behaviors; (4) gave the caregiver the toy to take home with a handout containing suggestions for other simple play activities to do at home; (5) encouraged caregivers to incorporate playtime with their child as often as possible and provided suggestions on how to integrate play into daily activities such as diaper changes and meal times to reinforce the importance of frequent parent–child play on their child’s development.	-	Shah et al. 2019 [40] (United States): Design: randomized static group comparison trial. Population: predominantly low-income caregivers of children aged 2–6 mo. Intervention: intervention families (*n* = 20) received SDP at recruitment and the subsequent well-child visit. Control families (*n* = 20) were provided handouts regarding developmental milestones. Administrators of SDP delivered the program during the waiting period in the examination room of the well-child visit. Assessment: StimQ-Infant used to assess parental behaviors that support children’s early cognitive development. The Parenting Sense of Competence Scale used to assess perceived parenting confidence. Results: SDP families scored 1.4 points higher on the Stim-Q Parental Involvement in Developmental Advance (*P* = 0.02). Although higher scores were noted for parent–child verbal interactions (*P* = 0.06) and provision of learning materials (*P* = 0.06) these scores were not statistically significant. No significant between-group differences on parenting self-efficacy and confidence. Shah et al., 2020 [41] (India): Design: prospective, noncomparative, mixed-methods feasibility study. Population: low-income caregivers of children aged 6 wk to 6 mo from rural villages in Karnataka, India, presenting for a well-child visit (*n* = 40). Assessment: implementation and practicality were operationalized as success with recruitment, ability to collect outcome measures, establishment of training and fidelity measures, and cost of the materials to deliver SDP. Acceptability and usefulness of the program were assessed through qualitative open-ended interviews. Results: parents found the intervention to be acceptable. Stimulation activities that promote early childhood development increased significantly among participants 1 mo after receipt of SDP (for example, told stories, *P* < 0.02; played with child, *P* < 0.01). The number of mothers, fathers, and nonparental caregivers who participated in 3 or more learning activities with their child over a 3-d period increased over the time of this study (*P* < 0.001). The strengths of this approach include alleviating the need for extra visits, delivery of content by administrators with a range of education, and optimizing all the time spent at a health care facility, including often wasted untapped time waiting for a provider. Suggestions for improvement from participants included more visual aids and wanting to receive SDP at multiple visits. Shah et al., 2022 [42] (India): Design: prospective cluster nonrandomized pilot and feasibility trial. Population: caregivers with infants aged 6–10 wk recruited from 2 primary health centers: one that delivered the intervention at 2 subsequent immunization visits (*n* = 25) and the other delivered care as usual (*n* = 28). Assessment: qualitative interviews and UNICEF Multiple Cluster Index Surveys items assessing quality of home stimulation and opportunities for learning. Results: number of caregivers who participated in 3+ learning activities with their child over a 3-d period increased significantly over the time of this study, favoring the intervention group (*P* < 0.005). Quality of home stimulation improved more in intervention than control group.
Care for Development Intervention (CDI)	Turkey	Ankara University School of Medicine	Outpatient clinic examination room At sick-child visits	< 24 mo	Materials: homemade toys Delivery agent: pediatrician Program description: promotion of child development at sick-child visits. The physicians listened for and observed positive caregiver–child interactions, used specific praise and positive reinforcement throughout the visit, and showed the caregiver examples of play and homemade toys for increased caregiver–child communication and age-appropriate stimulation. Recommended reading picture books to young children.	Standard CDI interview to assess how the caregiver played and communicated with the child in the home	Ertem et al., 2006 [43] (Turkey): Design: sequentially conducted controlled trial, with the comparison arm completed first. Intervention: the control arm (*n* = 113) received standard care. Intervention arm (*n* = 120) received CDI. Population: performed at an outpatient clinic setting at Ankara University School of Medicine, caregivers of children younger than 24 mo, enrolled in the study when they visited the clinic for minor illness or for a well-child visit Assessment: adapted version of Home Observation for Measurement of the Environment (HOME). Results: intervention groups had trend of more optimal HOME scores, more homemade toys were seen, and more caregivers reported reading to their child than in the comparison group.
Reach Out and Read	United States	Endorsed by American Academy of Pediatrics Designed by Boston Medical Center	Healthcare facility At well-child visits	6 mo to 5 y	Materials: age-appropriate children’s books. Delivery agents: pediatrician, healthcare provider, volunteer readers. Program description: healthcare providers were trained in techniques to promote the parents’ early literacy efforts with their children. This anticipatory guidance was incorporated into every well-child visit from 6 mo to 5 y. As part of these well-child checks, healthcare providers gave each child a developmental and culturally appropriate new book to take home to enrich the home-literacy environment. Volunteer readers were located in the waiting rooms of the clinical setting, providing literacy experiences for the children and modeling reading aloud for parents. Parents learn to be flexible when reading and to maximize interactive and dialogic reading.	-	Crosh et al., 2022 [44] (United States): Design: mixed methods study with quasi-experimental design. Population: caregivers of children aged 6 mo to 5 y recruited from outpatient clinic (the composition of the clinic at this time was 95% black, Latino/a, and other non-white identifying persons, and >90% of patients treated through the clinic had Medicaid insurance). Intervention: intervention families (*n* = 154) received books and guidance on book sharing at each well-child visit, control families received standard care (*n* = 50). Assessment: pre and postintervention survey measuring number of books in home, frequency, and perceived importance of book sharing. Results: parents in the intervention group reported significantly higher shared reading frequency and more children's books in the home compared with control group. Parents in the intervention group also reported higher importance and confidence in reading with their children. Other efficacy studies showing that Reach Out and Read promotes reading aloud and language development in children: Needlman et al., 1991 [45]; Jones et al., 2000 [46]; Sanders et al., 2000 [47]; Mendelsohn et al., 2001 [48]; Weitzman et al., 2004 [49].
Let's Read	Australia	Centre for Community Child Health at the Murdoch Children's Research Institute The Royal Children’s Hospital The Smith Family	Healthcare facility At well-child visits	4 mo to 3.5 y	Materials: age-appropriate books and other literacy promotion materials (for example, suggestions for interactive book-reading activities and lists of age-appropriate books). Delivery agent: Maternal and Child Health Nurses. Program description: 1 visit per year (2–10 min per visit), which included counseling and modeling regarding shared book-reading techniques. Provision of an age-appropriate book and other literacy promotion materials at each qualifying visit.	-	Goldfeld et al., 2011 [50] (Australia): Design: cluster randomized controlled trial. Population: infants attending their maternal and child health centers were recruited at age 1–2 mo from 5 relatively disadvantaged areas in Melbourne, Australia. Intervention: intervention group (*n* = 324) and control group (*n* = 228). The intervention (at 4, 8 to 12, 18, and 42 mo) comprised maternal and child health nurses modeling shared reading activities to parents, supported by parent information, and free books. Assessment: outcomes (at 2 y) included expressive vocabulary (MacArthur Bates Communicative Development Inventory), communication (Communication and Symbolic Behavior Scales), and home-literacy environment (StimQ-Toddler). Results: at 2 y of age, the intervention children and control children had similar scores on all levels. Deemed not to be successful in relatively disadvantaged communities.
Care for Child Development (CCD) Other names: Care for Child Development Intervention Care for Development (CFD)	Australia, Belize, Botswana, Brazil, Dominican Republic, El Salvador, India, Kazakhstan, Kenya, Kyrgyzstan, Mali, Mozambique, Pakistan, Paraguay, Peru, Tajikistan, Turkey, Uganda	WHO United Nations Children's Fund Extended partnership to national leaders and governments, development agencies, researchers, academics, nongovernmental organizations, professional associations, and advocacy groups	Healthcare facilities At well-child visits At sick-child visits Through home visits Through parenting groups ∗The chosen setting and frequency of contact are flexible	Birth to 5 y	Materials: counseling card, training manual and videos, facilitator guides for classroom activities and hands-on practice, presentations on its technical background and tools for monitoring and evaluation, children's homemade play toys, and ordinary household objects used for play. Delivery agent: CCD facilitators (can be doctors, nurses, daycare workers, social workers, or community health workers). Program description: an intervention model that seeks to strengthen the capacities of caregivers and families to promote children's development. Focuses on play for development, caregivers engaging with children often and with purpose, encourages father's involvement in caregiving, discusses milestones and child development that occurs without being able to see it. CCD facilitators interact with families at healthcare facilities in a variety of visits. Interactions include observing, coaching, and counseling, and providing positive encouragement for appropriate parenting practices.	-	Jin et al., 2007 [51] (China): Design: randomized trial. Population: families with a child aged < 2 y from 7 randomly selected villages in an impoverished rural county in China. Assessment: Gesell Developmental Schedules; questionnaire on family situation and knowledge, attitudes, and practices regarding child development. Results: after 6 mo, children in intervention group showed significantly higher adaptive, language, and social development quotients on the Gesell Development Schedules compared with control group. Responsive, rich interactions, and consistency in child rearing correlated with higher developmental scores. Gharehgoz et al., 2022 [52] (Iran): Design: quasi-experimental study. Population: mothers with children at risk of developmental delay in Tabriz, Iran. Age of children ranged from 4 to 36 mo. Fifty mothers selected through purposive sampling were then randomly assigned to either experimental or control groups (*n* = 25 each). Assessment: Social-Emotional Assessment/Evaluation Measure Family Profile (SEAM TM family profile) and Maternal Caregiving Quality Scale. Results: the CCD program was effective in promoting the competency of caregivers in all 3 variables of providing appropriate activities, predictable programs, and provision of play environment and safe home.
Bookstart	United Kingdom	BookTrust	Health facility Home visits	Birth to 12 mo	Materials: literacy packs including children's book, book mark, poem card, information about library resources, and information about the value of shared reading. Delivery agent: healthcare provider or health home visitors. Program description: nationwide program delivering literacy packs at either health clinics, home health visits, or at libraries to children between the ages of 6 and 9 mo. Since its inception, it has been expanded to offer further packs at key stages before school, as well as packs for children with additional needs, tips and guidance on reading together, resources, activities, and much more.	-	Wade and Moore, 1998 [53] (England): Design: quasi-experimental study, follow-up. Population: in cooperation with the school and parents of the original Bookstart children, researchers matched Bookstart children (now aged 5 y) based on 5 key characteristics to another child in the same class. Assessment: The Birmingham Baseline Assessment, includes 3 assessments made in English and 3 assessments made in mathematics. Results: Bookstart children were statistically significantly higher in reading and numbers. Bookstart children also produced higher speaking and listening scores, as well as writing and shape skills than comparison group (though not statistically significant). De Bondt and Bus, 2022 [54] (The Netherlands): Design: quasi-experimental study, follow-up. Population: original study enrolled children from age 8 mo to 15 mo from 35 health centers in the Netherlands. Experimental group received a Bookstart package (a infant case with 2 infant books and information about the importance of starting early with book reading). Comparison group did not receive Bookstart. A follow-up survey was conducted at age 5–6 y (*n* = 471). Assessment: language test, early literacy tests, and math tests from the children's Kindergarten. Results: Bookstart had no long-term effects on language and literacy skills in the general sample. In the 50% with the most reactive temperament in infancy, there was long-term improvements in language and literacy. There was no long-term impact of Bookstart on math skills. Bookstart families read significantly longer to their children.
Little by Little, WIC Program	United States	WIC	WIC Centers	Prenatal to 5 y	Materials: developmentally appropriate toys and books, informational handouts Delivery agent: WIC-trained staff Program description: at 4 visits a year, WIC families receive LBL services for each of their children. Brief counseling by WIC staff regarding child development tailored to child’s age. The counseling scripts differ according to child age, with the common theme of reading to children daily and interacting with them verbally whenever possible. Informational handout about child development milestones and positive parenting practices. The handouts include activities that do not require the purchase of additional toys or materials. Provision of books or developmentally appropriate toys for families to take home. Of the 22 items given to families from the prenatal period to age 5, 16 are books.	-	Whaley et al., 2011 [55] (United States): Design: quasi-experimental study Population: a control group (*n* = 200) received no intervention and an intervention group (*n* = 103) received 2 y of intervention; another additional intervention group (*n* = 102) received 4 y of the intervention. Participants and included parents of 3- to 4-y-old children. All families had low-income levels. Assessment: a single 2 h home visit that involved a school readiness assessment with the child, and assessment of the caregiver and home environment. Results: among Spanish speakers, the 4-y intervention group (*P* < 0.001) and the 2-y intervention group (*P* <0.05) had significantly higher school readiness scores, compared with the control group. Higher intervention exposure lead to higher home-literacy activities, which lead to better school readiness.

Abbreviations: CCD, Care for Child development; CDI, Care for Development Intervention; CFD, Care for Development; CRED, Control of Growth & Development for Infants and Children; GMP, growth, monitoring, and promotion; LBL, little by little; SDP, sit down and play.

Some of these interventions have been built into GMP programs at a national level, such as in Vanuatu and Peru. In Vanuatu, Save the Children, in collaboration with the Ministry of Health and the Ministry of Education, implemented the Bildimap Bren Blong Pikinini program (or GMP+), which integrated growth measurements and developmental milestone assessments to screen children for developmental delays or disabilities. On the basis of the results, children were either referred for further testing at provincial hospitals or caregivers were supported with encouragement and tailored activities to incorporate into their daily lives [56,57]. This program has not been evaluated for impact on ECD outcomes. The Control of Growth & Development for Infants and Children in Peru (CRED) program is delivered in health centers and supports parents in tracking their child's growth (both in terms of weight and height) and health and nutrition outcomes, and provides counseling to foster behavioral changes. UNICEF and the Government of Peru have incorporated Care for Child Development (CCD) into CRED [58]. CCD focuses on play for development, caregivers’ engagement with children (often and with purpose), encourages father's involvement in caregiving, and discusses milestones and child development with parents. At each CRED visit to the health center, nurses encourage play and child–caregiver interactions, recommend elements of responsive and sensitive feeding (for example, treating children as respectable individuals and communicating with children affectionately), and empower caregivers to provide nurturing care overall. Effectiveness of the program in improving functional outcomes in children (for example, developmental outcomes) has yet to be evaluated, but the program has proved successful in cultivating multisectoral engagement and sustained political commitment [58]. Despite Peru’s success, few other countries have implemented similar programs.

Other light-touch ECD interventions have been incorporated into sick or well-child visits, either in the clinic waiting room before starting the visit with the clinician [for example, playboxes in Mozambique, Sit Down and Play (SDP) in India] or during the visit (for example, CCD in Turkey and other countries, Reach Out and Read in the United States, Let’s Read in Australia, Bookstart in the United Kingdom, Healthy Steps for Young Children in the United States) (Table 3). In Mozambique, Program for Appropriate Technology in Health and the Ministry of Health instituted playboxes in healthcare facility waiting rooms. Playboxes included homemade picture books and toys, household objects, and posters of developmental milestones. Community volunteers were present in the waiting rooms to engage with parents and counsel those who struggled to interact with their children. Qualitative data from a 1-group pretest–posttest study indicated that the implementation of playboxes improved caregiver awareness and knowledge of developmental milestones, increased psychosocial stimulation of the children, and increased caregiver engagement during health facility consultations [39]. Outcomes on child development itself were not assessed. The SDP program in India was designed to be a short, low-cost program that incorporates key constructs of social cognitive theory to encourage positive parenting behaviors through take-home play activities. Sessions were held over a 10-min period although families waited in the examination room or in the waiting area. During the sessions, volunteers modeled play behaviors, engaged caregivers in discussion around developmental abilities and the importance of play, talk, and interaction, and provided encouragement and feedback to the caregivers on their interactions with the child. In randomized trials, SDP was found to improve the provision of psychosocial stimulation, but effects on child development have yet to be assessed [[40], [41], [42]]. In addition to its integration into CRED in Peru, the CCD intervention has been implemented in several low-income settings and through various locations, such as healthcare facilities, at well-child and sick-child visits, home visits, and through parenting groups. Randomized and quasi-experimental studies of CCD in low-income settings have shown benefits to child development and caregiver competencies [51,52]. Other interventions that have been similarly incorporated into well-child visits in high- or upper-middle-income countries include the Care for Development Intervention, Reach Out and Read, Let’s Read, and Bookstart (Table 3). Although mixed, evidence from effectiveness trials of these interventions in low-income families suggests some benefits to the quality and quantity of psychosocial stimulation received by children and parental self-efficacy around responsive caregiving behaviors [43,44,[51], [52], [53], [54]].

Lastly, the Little by Little School Readiness Program has been incorporated into the Women, Infants, and Children (WIC) program in the United States, through its own centers in certain locations. WIC is a United States federal assistance program targeting low-income pregnant women and mothers of children under 5. Little by Little targets children aged under 5 y. During 4 visits each year, caregivers receive counseling around ECD, developmental milestones, and positive parenting practices, and are provided with books or developmentally appropriate toys to take home. An evaluation of the program found that it improved home-literacy activities and school readiness [55].

In summary, a range of light-touch parenting interventions exist, many of them incorporating play-based activities that are similar to one another and have been implemented in countries from both the Global South and Global North. Only a few countries have already started implementing parenting interventions as part of GMP visits. Interventions that have not yet been implemented as part of GMP programs contain many elements that could feasibly be translated into GMP visits. Many of them, however, have not been evaluated in terms of their impacts on ECD.

## Discussion

We investigated whether the GMP platform can be used to improve ECD in low- and middle-income settings. We show that growth indices are not accurate predictors of concurrent or later children’s developmental abilities, and therefore, should not be used to identify children at risk of suboptimal development at either the individual or group levels. Rather, if the objective is to screen for developmental delays, specialized tools should be used. A review of short and simple tools indicated that the ASQ or the SWYC could be used by frontline health workers in the context of GMP visits, including in low- and middle-income countries. Lastly, a review of light-touch ECD interventions that could be feasibly incorporated into GMP visits identified a wide range of effective interventions. Most effective interventions were those in which the service provider demonstrated an activity with the child, gave time to the caregiver to practice the activity, and provided encouragement and feedback.

Although growth indices have been associated with child development scores [11,25], their utility as predictors of which individual children are at risk of developmental delays has never been demonstrated. Our analyses indicated that growth indices and growth indicators are not accurate predictors of how the individual child is performing, or will perform, developmentally. Alternatively, several caregivers report developmental screening tools exist which could feasibly be incorporated into GMP visits. Tools focusing on development milestones alone are insufficient to identify children who are at risk of suboptimal development, however, because assessments of milestones often have low predictive validity and their validity varies across age ranges [59,60]. Assessing caregiver concerns rather than children’s skills or abnormal patterns of development, using a tool such as the SWYC or the PEDS, can result in more caregiver investment in and attention to the child’s development [61].

Identifying individual children at risk of delay, however, is unnecessarily complicated and expensive when trying to improve the developmental potential of children in resource-limited settings. Instead, in low-resource settings where many children face suboptimal psychosocial stimulation and other adverse contextual conditions that lead to reduced developmental outcomes, a public health strategy of intervening on all children may be warranted. This type of universal approach has been shown to be more effective than targeting at-risk children with, for instance, food assistance to alleviate child undernutrition in resource-limited settings [62].

Another consideration is whether the outcomes of developmental assessments are used to guide intervention. An important issue arises in settings where screening for delays or disabilities does not result in actions or plans to address them because either there are no nearby facilities or professionals, or these services are unaffordable to families. Identifying developmental concerns without a plan to address them is unproductive and unethical. Rather, all children in resource-limited settings, regardless of their scores on screening tests, can benefit from receiving messaging around early learning and responsive parenting, and we identified several of these types of interventions that are feasible to implement in resource-constrained settings.

Early learning and parenting programs, even light-touch versions, should include the promotion of responsive caregiving. The feasibility of this strategy was demonstrated by the Peru CRED program and CCD. Programs that promote responsive caregiving are more effective in improving child cognitive development and parent–child interactions and show 4-fold larger effects on parenting practices compared with programs that do not include responsive caregiving content [63]. Beyond the promotion of early play and the provision of age-appropriate toys and manipulatives, ECD programs should also focus on promoting interactions that are responsive, emotionally supportive, and developmentally stimulating [64,65]. Furthermore, programs should also support and enable caregivers to engage with their children by addressing parental stress and depression [66,67]. In some cases, intensive programs that promote early learning and responsive parenting, such as CCD, have been adapted and condensed so that they could be delivered as short, light-touch versions of the program during well-child visits [43,51,52]. How to optimally implement these types of programs, including the number and frequency of visits and skills necessary to deliver the program appropriately, remains unclear and requires further study [68].

Our study investigated the potential of using the GMP platform to identify children at risk of suboptimal development and deliver early learning and responsive parenting interventions, with the goal of improving child development. Growth, which is currently measured as part of GMP programs across the world, is not an accurate predictor of the developmental status of a child and should not be used to identify children at risk of developmental delays. Screening tools are available to identify children at risk of suboptimal development, and some could feasibly be built onto GMP visits where identifying developmental delays is a priority. Nevertheless, given the high potential of most (or all) children living in resource-limited settings to benefit from caregiving interventions and the challenges of measuring each child’s development, universal targeting of caregiving interventions is likely to be more cost-effective than attempting to identify and target specific at-risk children. Light-touch parenting interventions have been delivered by frontline health workers in low- and high-income country settings, but few have been evaluated in terms of their effectiveness on child development outcomes. There is an urgent need for evaluation and implementation research to assess which ECD models and context-specific implementation approaches are most effective in improving child development outcomes in the context of GMP and to address potential implementation constraints when delivering ECD services through GMP programs in low- and middle-income country settings.

## Author contributions

The authors’ contributions were as follows – LML, EAF, MTR, JLL: designed the research; LML, FA, SW: conducted the analyses and reviewed the literature; LML: drafted the manuscript; and all authors: critically reviewed the manuscript and read and approved the final manuscript.

## Data availability

The findings of this analysis were supported by previously combined datasets from EAF. The data are available, upon reasonable request, to the authors.

## Funding

This research was supported by the Bill & Melinda Gates Foundation (INV-042766) through an award to the International Food Policy Research Institute. The funder had no role in the study design; in the collection, analysis, and interpretation of data; in the writing of the report; and in the decision to submit the paper for publication.

## Conflict of interest

JLL reports financial support was provided by Bill and Melinda Gates Foundation. Other authors declare they have no known competing financial interests or personal relationships that could have appeared to influence the work reported in this paper.
