# Supplementary material for: Perspective: Growth Monitoring and Promotion as an Opportunity to Improve Early Childhood Development
Source: Adv Nutr. 2025 Jun 25;16(8):100470. doi: 10.1016/j.advnut.2025.100470 (PMC12296435; doi:10.1016/j.advnut.2025.100470)
Supplement: multimedia component 1 [file mmc1.docx]

Supplementary Data for

**Perspective: Growth monitoring and promotion as an opportunity to improve early childhood development**

**Authors**

Leila M Larson,^1^ Edward A. Frongillo,^1^ Fahmida Akter,^1^ Shelbie Wooten,^1^ Rebecca Brander,^2^ Marie Ruel,^2^ Jef L. Leroy^2^

Corresponding author. Email: [larsonl@mailbox.sc.edu](mailto:larsonl@mailbox.sc.edu)

**Supplemental Methods**

To construct the MICS4 wealth index, principal components analysis was performed by using information on the ownership of consumer goods, dwelling characteristics, water and sanitation, and other characteristics that are thought to relate to the household’s wealth, such as persons per room, access to internet, and energy use, to generate weights (factor scores) for each of the items used. Factor scores were calculated for the total sample. Each household in the total sample was then assigned a wealth score based on the assets owned by that household and on the factor scores obtained. The survey household population was then ranked according to the wealth score of the household they were living in and was finally divided into 5 equal parts (quintiles) from lowest (poorest) to highest (richest). The wealth index is assumed to capture the underlying long-term wealth through information on the household assets and is intended to produce a ranking of households by wealth, from poorest to richest. It does not provide information on absolute poverty, current income, consumption, or expenditure levels.

| **Supplemental Table 1**: AUCs for HAZ, WAZ, and WHZ's ability to discriminate children at risk of poor developmment, using MINIMat data (N=2804)* | | | |
| --- | --- | --- | --- |
| **Dependent variable (score ≥ the cutoff for):** | **AUC (Bayley cutoff of 70)** | **AUC (Bayley cutoff of 80)** | **AUC (Bayley cutoff of 85)** |
| **Independent variable: Height for age z-score** | | | |
| Psychomotor developmental Index (PDI) at 7 months | 0.55 | 0.56 | 0.57 |
| Psychomotor developmental Index (PDI) at 18 months | 0.55 | 0.56 | 0.56 |
| Mental Developmental Index (MDI) at 18 months | 0.57 | 0.60 | 0.60 |
|  |  |  |  |
| **Independent variable: Weight for age z-score** | | | |
| Psychomotor developmental Index (PDI) at 7 months | 0.55 | 0.56 | 0.57 |
| Psychomotor developmental Index (PDI) at 18 months | 0.52 | 0.53 | 0.54 |
| Mental Developmental Index (MDI) at 18 months | 0.54 | 0.58 | 0.58 |
|  |  |  |  |
| **Independent variable: Weight for height z-score** | | | |
| Psychomotor developmental Index (PDI) at 7 months | 0.53 | 0.52 | 0.53 |
| Psychomotor developmental Index (PDI) at 18 months | 0.50 | 0.50 | 0.51 |
| Mental Developmental Index (MDI) at 18 months | 0.51 | 0.55 | 0.54 |
| *AUC, area under the receiver operating characteristic curves; HAZ, height-for-age z-score; MDI, mental development index; PDI, psychomotor development index; WAZ, weight-for-age z-score; WHZ, weight-for-height z-score. | | | |

| **Supplemental Table 2**: AUCs for individual level HAZ, WAZ, and WHZ's ability to discriminate individual children not at-risk of suboptimal development, using MICS4 data* | | | | | | | | |
| --- | --- | --- | --- | --- | --- | --- | --- | --- |
|  | **Independent variable: HAZ** | |  | **Independent variable: WAZ** | |  | **Independent variable: WHZ** | |
| **Dependent variable (adequacy):** | **N** | **AUC** |  | **N** | **AUC** |  | **N** | **AUC** |
| Literacy numeracy | 69429 | 0.63 |  | 70410 | 0.60 |  | 68596 | 0.51 |
| Learning | 68693 | 0.60 |  | 69635 | 0.61 |  | 67858 | 0.54 |
| Social-emotional | 68577 | 0.59 |  | 69531 | 0.57 |  | 67754 | 0.50 |
| Physical | 69370 | 0.54 |  | 70356 | 0.54 |  | 68525 | 0.52 |
| Total ECDI | 67081 | 0.60 |  | 67999 | 0.59 |  | 66304 | 0.53 |
| *ECDI, Early Child Development Index; HAZ, height-for-age z-score; WAZ, weight-for-age z-score; WHZ, weight-for-height z-score. | | | | | | | | |

| **Supplemental Table 3**: Estimates of cluster-level associations between growth indices and child development domains, using MICS4 data* | | | | | | | | | | | | | | | | | | |
| --- | --- | --- | --- | --- | --- | --- | --- | --- | --- | --- | --- | --- | --- | --- | --- | --- | --- | --- |
|  |  | **Independent variable: Cluster mean HAZ** | | | | |  | **Independent variable: Cluster mean WAZ** | | | | |  | **Independent variable: Cluster mean WHZ** | | | | |
| **Dependent variable (cluster proportion of children with adequate):** | **Average cluster proportion of children with adequate development (%)** | **SD** | **RMSE** | **Regression coefficient** | **SE** | **P-Value** |  | **SD** | **RMSE** | **Regression coefficient** | **SE** | **P-Value** |  | **SD** | **RMSE** | **Regression coefficient** | **SE** | **P-Value** |
| Literacy numeracy | 23.0 | 0.81 | 0.30 | 0.06 | 0.002 | <.0001 |  | 0.81 | 0.30 | 0.07 | 0.003 | <.0001 |  | 0.81 | 0.30 | 0.02 | 0.003 | <.0001 |
| Learning | 88.1 | 0.37 | 0.20 | 0.05 | 0.002 | <.0001 |  | 0.37 | 0.20 | 0.06 | 0.002 | <.0001 |  | 0.37 | 0.20 | 0.03 | 0.002 | <.0001 |
| Social-emotional | 76.4 | 0.44 | 0.27 | 0.03 | 0.002 | <.0001 |  | 0.44 | 0.26 | 0.04 | 0.002 | <.0001 |  | 0.44 | 0.27 | 0.03 | 0.002 | <.0001 |
| Physical | 96.6 | 0.19 | 0.11 | 0.01 | 0.001 | <.0001 |  | 0.19 | 0.11 | 0.01 | 0.001 | <.0001 |  | 0.19 | 0.11 | 0.00 | 0.001 | 0.5549 |
| Total ECDI | 72.2 | 0.47 | 0.29 | 0.07 | 0.002 | <.0001 |  | 0.47 | 0.29 | 0.09 | 0.003 | <.0001 |  | 0.47 | 0.29 | 0.05 | 0.003 | <.0001 |
| * Units for the SD, RMSE, regression coefficient, and SE are the same as the dependent variables. ECDI, Early Child Development Index; HAZ, height-for-age z-score; RMSE, root mean square error; WAZ, weight-for-age z-score; WHZ, weight-for-height z-score. | | | | | | | | | | | | | | | | | | |

| **Supplemental Table 4**: Estimates of cluster-level associations between growth indicators and child development domains, using MICS4 data* | | | | | | | | | | | | | | | | | | |
| --- | --- | --- | --- | --- | --- | --- | --- | --- | --- | --- | --- | --- | --- | --- | --- | --- | --- | --- |
|  |  | **Independent variable: cluster prevalence of stunting** | | | | |  | **Independent variable: cluster prevalence of wasting** | | | | |  | **Independent variable: cluster prevalence of underweight** | | | | |
| **Dependent variable (cluster proportion of children with inadequate):** | **Average cluster proportion of children with inadequate development (%)** | **SD** | **RMSE** | **Regression coefficient** | **Standard Error** | **P-Value** |  | **SD** | **RMSE** | **Regression coefficient** | **Standard Error** | **P-Value** |  | **SD** | **RMSE** | **Regression coefficient** | **Standard Error** | **P-Value** |
| Literacy numeracy | 77.0 | 0.81 | 0.30 | 0.21 | 0.009 | <.0001 |  | 0.81 | 0.30 | 0.04 | 0.013 | 0.0008 |  | 0.81 | 0.30 | 0.20 | 0.011 | <.0001 |
| Learning | 11.9 | 0.37 | 0.20 | 0.20 | 0.006 | <.0001 |  | 0.37 | 0.20 | 0.12 | 0.009 | <.0001 |  | 0.37 | 0.20 | 0.24 | 0.008 | <.0001 |
| Social-emotional | 23.6 | 0.44 | 0.27 | 0.11 | 0.008 | <.0001 |  | 0.44 | 0.27 | 0.06 | 0.011 | <.0001 |  | 0.44 | 0.27 | 0.14 | 0.010 | <.0001 |
| Physical | 3.4 | 0.19 | 0.11 | 0.04 | 0.003 | <.0001 |  | 0.19 | 0.11 | 0.02 | 0.004 | <.0001 |  | 0.19 | 0.11 | 0.05 | 0.004 | <.0001 |
| Total ECDI | 27.8 | 0.47 | 0.29 | 0.27 | 0.009 | <.0001 |  | 0.47 | 0.30 | 0.12 | 0.012 | <.0001 |  | 0.47 | 0.29 | 0.31 | 0.011 | <.0001 |
| * Units for the SD, RMSE, regression coefficient, and SE are the same as the dependent variables. ECDI, Early Child Development Index; RMSE, root mean square error. | | | | | | | | | | | | | | | | | | |

| **Supplemental Table 5**: Estimates of cluster-level associations between growth indices, maternal education, and wealth with child development domains, using MICS4 data* | | | | | |
| --- | --- | --- | --- | --- | --- |
| **Dependent variable (cluster proportion of children with adequate):** | **Independent variables (cluster mean)** | **RMSE** | **Regression coefficient** | **SE** | **P-value** |
| Literacy numeracy | HAZ | 0.28 | 0.02 | 0.003 | <.0001 |
|  | WAZ |  | 0.01 | 0.004 | 0.0006 |
|  | Maternal education |  | 0.05 | 0.003 | <.0001 |
|  | Wealth index |  | 0.06 | 0.002 | <.0001 |
| Learning | HAZ | 0.20 | 0.01 | 0.002 | <.0001 |
|  | WAZ |  | 0.03 | 0.002 | <.0001 |
|  | Maternal edu |  | 0.05 | 0.002 | <.0001 |
|  | Wealth index |  | -0.01 | 0.001 | <.0001 |
| Social-emotional | HAZ | 0.26 | 0.00 | 0.003 | 0.1749 |
|  | WAZ |  | 0.03 | 0.003 | <.0001 |
|  | Maternal education |  | 0.04 | 0.003 | <.0001 |
|  | Wealth index |  | -0.01 | 0.002 | <.0001 |
| Physical | HAZ | 0.10 | 0.01 | 0.001 | <.0001 |
|  | WAZ |  | 0.00 | 0.001 | 0.0405 |
|  | Maternal education |  | 0.01 | 0.001 | <.0001 |
|  | Wealth index |  | 0.00 | 0.001 | 0.0891 |
| Total ECDI | HAZ | 0.28 | 0.02 | 0.003 | <.0001 |
|  | WAZ |  | 0.04 | 0.004 | <.0001 |
|  | Maternal education |  | 0.08 | 0.003 | <.0001 |
|  | Wealth index |  | 0.01 | 0.002 | 0.0013 |
| *The wealth index used quintiles, from poorest to richest. Maternal education categorized from 0 to 9, representing increasing years of schooling. Units for the RMSE, regression coefficient, and SE are the same as the dependent variables. ECDI, Early Child Development Index; HAZ, height-for-age z-score; RMSE, root mean square error; WAZ, weight-for-age z-score; WHZ, weight-for-height z-score. | | | | | |

| **Supplemental Table 6**: Estimates of cluster-level associations between growth indicators, maternal education, and wealth with child development domains, using MICS4 data* | | | | | |
| --- | --- | --- | --- | --- | --- |
|  |  |  |  |  |  |
| **Dependent variable (cluster proportion of children with inadequate):** | **Independent variables (cluster proportion)** | **RMSE** | **Regression coefficient** | **SE** | **P-value** |
| Literacy numeracy | Stunting | 0.28 | 0.08 | 0.012 | <.0001 |
|  | Underweight |  | -0.04 | 0.016 | 0.0212 |
|  | No maternal education | | 0.12 | 0.008 | <.0001 |
|  | Poorest wealth quintile | | -0.07 | 0.002 | <.0001 |
| Learning | Stunting | 0.20 | 0.09 | 0.009 | <.0001 |
|  | Underweight |  | 0.07 | 0.011 | <.0001 |
|  | No maternal education | | 0.11 | 0.005 | <.0001 |
|  | Poorest wealth quintile | | 0.00 | 0.001 | 0.9491 |
| Social-emotional | Stunting | 0.27 | 0.04 | 0.012 | 0.0019 |
|  | Underweight |  | 0.03 | 0.015 | 0.0661 |
|  | No maternal education | | 0.08 | 0.007 | <.0001 |
|  | Poorest wealth quintile | | 0.00 | 0.002 | 0.2029 |
| Physical | Stunting | 0.11 | 0.03 | 0.005 | <.0001 |
|  | Underweight |  | 0.01 | 0.006 | 0.0454 |
|  | No maternal education | | 0.01 | 0.003 | 0.0007 |
|  | Poorest wealth quintile | | 0.00 | 0.001 | <.0001 |
| Total ECDI | Stunting | 0.28 | 0.11 | 0.013 | <.0001 |
|  | Underweight |  | 0.05 | 0.016 | 0.0019 |
|  | No maternal education | | 0.17 | 0.008 | <.0001 |
|  | Poorest wealth quintile | | -0.02 | 0.002 | <.0001 |
| * Units for the RMSE, regression coefficient, and SE are the same as the dependent variables. ECDI, Early Child Development Index; RMSE, root mean square error. | | | | | |

**Supplemental Figure 1**: Estimated coefficient of association between growth indices and Bayley scores by quantile level, MINIMat study^1^


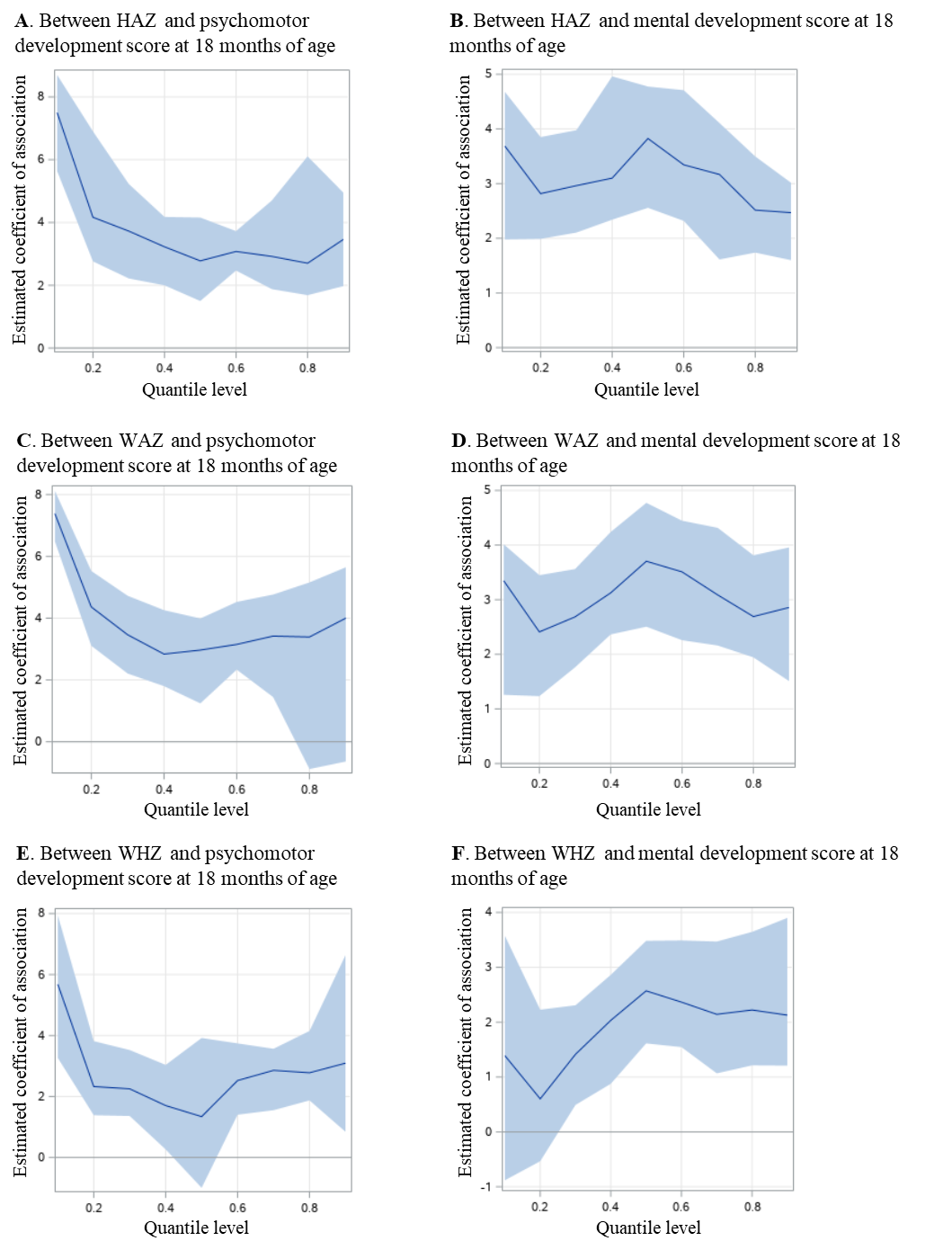


^1^Solid line represents the coefficient of association and blue shaded area represents the 95% CI. HAZ: height-for-age z-score; WAZ: weight-for-age z-score; WHZ, weight-for-height z-score.
